# In silico study on Penicillin derivatives and Cephalosporins for upper respiratory tract bacterial pathogens

**DOI:** 10.1007/s13205-013-0147-z

**Published:** 2013-06-11

**Authors:** K. M. Kumar, P. Anitha, V. Sivasakthi, Susmita Bag, P. Lavanya, Anand Anbarasu, Sudha Ramaiah

**Affiliations:** School of Biosciences and Technology, VIT University, Vellore, 632014 Tamil Nadu India

**Keywords:** Upper respiratory tract infections, Penicillin binding proteins, β-Lactam antibiotics, Docking

## Abstract

Upper respiratory tract infection (URTI) is an acute infection which involves the upper respiratory tract: nose, sinuses, tonsils and pharynx. URT infections are caused mainly by pathogenic bacteria like *Streptococcus pneumoniae, Haemophilus influenzae and Staphylococcus aureus.* Conventionally, β-lactam antibiotics are used to treat URT infections. Penicillin binding proteins (PBPs) catalyze the cell wall synthesis in bacteria. β-Lactam antibiotics like Penicillin, Cephalosporins, Carbapenems and Monobactams inhibit bacterial cell wall synthesis by binding with PBPs. Pathogenic bacteria have efficiently evolved to resist these β-lactam antibiotics. New generation antibiotics are capable of inhibiting the action of PBP due to its new and peculiar structure. New generation antibiotics and Penicillin derivatives are selected in this study and virtually compared on the basis of interaction studies. 3-Dimensional (3D) interaction studies between Lactivicin, Cefuroxime, Cefadroxil, Ceftaroline, Ceftobiprole and Penicillin derivatives with PBPs of the above-mentioned bacteria are carried out. The aim of this study was to suggest a potent new generation molecule for further modification to increase the efficacy of the drug for the URTI.

## Introduction

The respiratory tract is a frequent site of infection because it comes in direct contact with the physical environment and is exposed to airborne microorganisms. Worldwide, approximately 4 million children under 5 years of age die each year from respiratory tract infections (RTIs) (Garenne et al. 1992). It is estimated that throughout the world 1.9 million children <5 years old died from acute respiratory infection in 2001, 70 % of them in Africa and South East Asia (Williams et al. 2001). Nasopharyngitis, pharyngitis, tonsillitis and otitis media are common upper respiratory tract (URT) infections which constitute 87.5 % of the total episodes of respiratory infections. URT infections can be caused by a variety of bacteria like *Chlamydia pneumoniae, Mycoplasma pneumoniae, Streptococcus pyogenes, Streptococcus pneumoniae, Bordetella pertussis, Staphylococcus aureus, Escherichia coli* and *Haemophilus influenzae* (Peter et al. 1985). The majority of URT infections are caused by only three species *S. pneumoniae*, *S. aureus* (Gram-positive bacteria) and *H. influenzae* (Gram-negative bacteria). The treatments of these three bacterial infections have been more complicated by the emergence and spread of multi-drug resistant strains (Doern et al. 1988, 1997). Two mechanisms have been reported to be responsible for antibiotic resistance: structural modification in Penicillin binding protein (PBP) targets and production of β-lactamase, first identified in 1972 (Williams and Moosdeen 1986; Reid et al. 1987; Jorgensen 1992). PBPs are the membrane bound enzymes which catalyze the steps involved in bacterial cell wall biosynthesis and are the target enzymes of β-lactam antibiotics (Ghuysen 1991; Goffin and Ghuysen 1998; Macheboeuf et al. 2006; Sauvage et al. 2008). Peptidoglycan is the major component of bacterial cell wall synthesized by PBPs. Every bacterial species has more than two PBPs. *S. pneumoniae*, the major human pathogen causing URT infections is responsible for over 1.6 million deaths every year (Lynch and Zhanel 2005). It has six PBPs, PBP1a, PBP1b, PBP2a, PBP2b, PBP2x and PBP3, which are highly conserved. Penicillin resistance in *S. pneumoniae* has been reported in many countries. The mechanism of Penicillin resistance is due to the modification of active site motif in PBPs of *S. pneumoniae*. Penicillins and extended spectrum Cephalosporins have high level of resistance to PBP1a, PBP2x and PBP2b of *S. pneumoniae* (Sheldon and Mason 1998). *S. aureus* is a potent pathogen that can cause respiratory tract infections (Ragle et al. 2010). It has PBP1b, PBP2a and PBP3. The resistance of *S. aureus* to Penicillin was identified in 1940 and 1965, but recently it has become a major threat to public health concern (Metan et al. 2005), alteration in PBP2a encoded gene decreases the affinity of most β-lactam antibiotics. *H. influenzae* is a common and exclusively human commensal of the nasopharynx. *H. influenzae* colonizes in the nasal cavity of approximately 80 % of the human population. *H. influenzae* has PBP4 and PBP5 which are low molecular weight proteins. The treatment of *H. influenzae* infections has been more complicated by the emergence and spread of multi-drug resistant strains (Doern et al. 1988, 1997). Several computational investigations have been done on β-lactam antibiotics and PBPs. Yoshida et al. reported the crystal structures of PBP3 in methicillin-resistant *S. aureus* (MRSA) and nature of its interactions with Cefotaxime. The study explains in detail about the hydrophobic and hydrogen bond interaction of Cefotaxime with the active sites of the PBP3 and PBP2 of *S. aureus*. Experimentally they proved it with nanoelectrospray mass spectrometry and ultracentrifugation to measure its sensitivity to different types of Penicillin derivatives (Yoshida et al. 2012). Samo Turk et al. study mainly focused to discover non-covalent inhibitor for PBP2x and PBP2a experimentally and computationally. The study reported the minimum inhibitory concentration of non-covalent inhibitor against several Gram-positive bacterial strains, including MRSA and analyzed the binding affinity of inhibitor with PBP2a and PBP2x (Turk et al. 2011). Another computational study investigated the interaction of Carbenicillin, Ceftazidime and Cefotaxime with binding site of PBP1b and PBP3 (Sainsbury et al. 2011). Sainsbury et al. reported the crystal structures of apo-PBP and complexes with Ceftazidime and Carbenicillin and investigated the similarities and differences between these structures. Fumihiro Kawai et al. determined the high-resolution apo crystal structures of two-low molecular weight PBPs, PBP4 and PBP5 from *H. influenzae.* They demonstrated the binding affinity of designed β-lactam antibiotics and Amoxicillin with PBP4 and PBP5 (Kawai et al. 2010). Though Penicillin derivatives and Cephalosporins have been used for bacterial infections over a period of time, many bacterial pathogens have become resistant to these antibiotics. One major mode of resistance is by the alternation of PBPs resulting in low affinity to β-lactam antibiotics. Researchers have explored the mechanism of resistance to β-lactam antibiotics using only a few Penicillin derivatives or Cephalosporins (Sainsbury et al. 2011; Turk et al. 2011; Yoshida et al. 2012). This prompted us to investigate in detail using a wide spectrum of β-lactam antibiotics (both Penicillin derivatives and Cephalosporins). Our results indicate that of 19 β-lactam antibiotics, Ceftobiprole and Ceftaroline might have better affinity to PBPs and hence it may be effective in the treatment of URT bacterial infections. Our results are also comparable to previous experimental findings (Hebeisen et al. 2001; Sader et al. 2005; Jones et al. 2005; Kosowska et al. 2005; Davies et al. 2006; Citron and Goldstein 2008; Estrada et al. 2008; Henry et al. 2010; Kosowska et al. 2010; Mosian et al. 2010; Dauner et al. 2010) and the findings of our research might provide clues as to how Ceftobiprole and Ceftaroline exert their inhibitory action on bacterial pathogens.

## Methods

### Preparation of macromolecular and small molecular models

PBP was thought to be essential for the synthesis of bacterial cell wall. All types of the PBPs (PBP1a, PBP1b, PBP2a, PBP2b, PBP2x, PBP3, PBP4, PBP5 and PBP6) were selected for this study. 3-Dimensional (3D) structures of the PBPs were obtained from Protein Data Bank (PDB) (Berman et al. 2000). 3D structures of PBPs were visualized through PyMOL viewer (Lill and Danielson 2010). Co-crystallized ligands were identified and removed from the target proteins then water molecules removed and H atoms were added to the structure and minimizations were performed using Swiss pdb viewer (Guex and Peitsch 1997). The 3D coordinates of the Penicillin derivatives and Cephalosporins were obtained from NCBI PubChem Compound database (Li et al. 2010) and constructed using chemsketch (Li et al. 2004). Hydrogen atoms were added to all the structures and gasteiger atomic partial charges were computed. A geometry optimization of all the compounds was performed using chimera (Pettersen et al. 2004) for flexible conformations of the compounds during the docking.

**Fig. 1:**
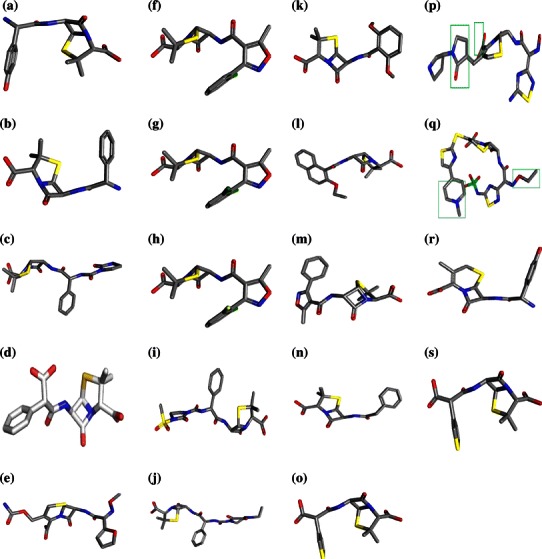
3-Dimensional structures of Penicillin derivatives and Cephalosporins: **a** Amoxicillin, **b** Ampicillin, **c** Azlocillin, **d** Carbenicillin, **e** Cefuroxime, **f** Cloxacillin, **g** Dicloxacillin, **h** Flucloxacillin, **i** Mezlocillin, **j** Piperacillin, **k** Methicillin, **l** Nafcillin, **m** Oxacillin, **n** Penicillin G, **o** Ticarcillin, **p** Ceftobiprole, **q** Ceftaroline, **r** Cefadroxil and **s** Lactivicin (The *highlighted boxes* indicate the non-essential components in **p** Ceftobiprole and **q** Ceftaroline respectively)

PDB ID of every PBP was depicted in Table 1 and two-dimensional structures of Penicillin derivatives and Cephalosporins are shown in Fig. 1.

**Table 1:** Active site residues of PBPs

PBPs	PDB ID	Name of the organism	Active site residues
PBP1a	2C6W	*Streptococcus* *pneumoniae*	Ala270, Tyr271, Asp273, Asn274, Trp311, Asn315, Leu345, Gly346, Ala347, Arg348, His349, Hln350, Ser351
PBP1b	2Y2Q	*Staphylococcus aureus*	Asp337, Phe341, Thr342, Ala345, Glu346, Glu349, Tyr443, Gln447, Asn448, Asn449, Phe452, Asp453, Glu540
PBP2a	1VQQ	*Staphylococcus aureus*	Ser403, Lys406, Arg445, Tyr446, Glu447, Ile459, Glu460, Ser403, Ser462, Asp463, Asn464
PBP2b	2WAE	*Streptococcus pneumoniae*	Thr55, Thr56, Ser57, Ser81, Gln180, Ala183, Val184, Gly185, Ala188, Thr189, Gly190, Thr191, Ser218, Ser258, Leu259, Asn260, Asp261, Arg 262, Arg280
PBP2x	1PYY	*Streptococcus pneumoniae*	Lys420,Val423, Pro424, Thr425, Arg426, Arg463, Glu476, Glu497, Ile498, Val499, Gly500, Ala650, Arg654, Pro660, Ile661, Val662, Gly664
PBP3	3OC2	*Streptococcus pneumoniae*	Ala162, His163, Gly166, Phe167, Arg175, Glu176, Gly177, Leu180, Tyr268, Pro278, Met281, Arg282, Asn283, Met286, Ile287, Phe383, Pro384, Gly385, Glu386, Arg387
PBP4	1TVF	*Staphylococcus aureus*	Gln133, Val136, Ser137, Asn138, Ser139, Phe225, Phe225, Thr226, Lys227, Gln228, Tyr239, Thr240, Phe241, Asn242, Leu245, Leu258, Lys259, Thr260
PBP5	3A3J	*Haemophilus influenzae*	Val75, Val77, Leu79, Lys80, Asn86, Asn121, Asp193, Leu194, Leu194, Pro195, Glu196, Glu197, Ile200
PBP6	3ITB	*Escherichia coli*	Ser40, Ile103, Ile104, Gln105, Ser106, Pro192, Asn193, Arg194, Asn195, Met208, Lys209, Thr210, Gly211, Thr212

### Active site identification

The catalytic binding site was believed to be a small region, a cleft or pocket, where lead molecules can bind to stimulate the target protein and produce the desirable effect. Thus, recognizing the catalytic binding site residues in the protein structure was of high importance in computer-aided drug designing. Identification of accurate catalytic binding site was difficult because the target proteins were capable of undergoing conformational changes (Liao and Andrews 2007). Qsite finder (Laurie and Jackson 2005) recognizes the possible ligand binding sites using the van der Waal’s probes and interaction energy. In the present study, Qsite finder was employed for locating the active sites in PBP1a, PBP1b, PBP2a, PBP2b, PBP2x, PBP3, PBP4, PBP5 and PBP6 proteins.

### Virtual screening of β-lactam antibiotics

iGEMDOCK (A Generic Evolutionary Method for molecular DOCKing) automated docking program (Yang and Chen 2004). iGEMDOCK integrated the structure-based virtual screening, molecular docking, post screening analysis and visualization steps. We selected all types of PBPs (PBP1a, PBP1b, PBP2a, PBP2b, PBP2x, PBP3, PBP4, PBP5 and PBP6) to carry out the structure-based virtual screening study of penicillin derivatives and Cephalosporins. The 3D coordinates of each therapeutic target protein and ligand molecules were implemented through the GEMDOCK graphical environment interface. Before docking, the output path was set. GEMDOCK default parameters included the population size (*n* = 200), generation (*g* = 70) and number of solutions (*s* = 10) to compute the probable ligand binding mechanism for each target protein. Then the docking run was started using GEMDOCK scoring function. After docking, the individual binding pose of each ligand was observed and their binding affinity with the target proteins was analyzed. In the post docking screening the best binding pose and total energy of each ligand was analyzed. The details of best binding pose and total energy values were saved in output folder. Protein–ligand binding site was analyzed and visualized using PyMOL (Lill and Danielson 2010).

### Docking

The automated docking studies were carried out using Auto-Dock version 4.0 (Morris et al. 2009). 3D structure of each PBPs were implemented through the graphical user interface AUTODOCKTOOLS (ADT 1.4.6). The graphical user interface AUTODOCKTOOLS was performed to set up the enzymes: all hydrogens were added, Kollman United Atoms charges loaded and non-polar hydrogens were merged to carbon atoms. The initial parameters and van der Waals well depth of 0.100 kcal/mol for macromolecules, generated PDBQT files were saved. The 3D structures of ligand molecules were constructed, optimized, and converted into Mol2 file format with the help of the chimera. The charges of the non-polar hydrogen atoms are assigned to the atom to which the hydrogen is attached. The resulting files were saved as PDBQT files. The drug binding site for the ligands on PBP1a, PBP1b, PBP2a, PBP2b, PBP2x, PBP3, PBP4, PBP5 and PBP6 were identified using Qsite finder online server. The grid point was set at the ligand binding site in each one of the obtained PDB structures. AUTODOCK 4.0 was performed for all docking calculations. The AUTODOCKTOOLS was used to generate the grid parameter files and docking parameter files. The docking parameters were also used to calculate docking scores for β-lactam antibiotics and Penicillin derivatives. Protein–ligand docking calculations were carried out on PBPs. Lamarckian genetic algorithm (Morris et al. 1998) was used to generate possible protein–ligand binding conformations.

### ADME screening

The molinspiration (Jarrahpour et al. 2011) server was used to predict the ADME properties of the antibiotics. It predicted both physiochemical and pharmacological properties. Smiles (Simplified Molecule Input Line Entry Specification) of the antibiotics was submitted. It predicted the properties of the drug such as molecular volume, number of hydrogen bond donors and acceptors, LogP and rotatable bonds. It provided high-speed molecular properties calculated and drug likeness for a given compound. The acceptability of the analogs is evaluated based on Lipinski’s rule of 5 (Lipinski et al. 2006), which is essential for structure-based drug design.

## Results and discussion

The 3D structures of PBP1a, PBP1b, PBP2a, PBP2b, PBP2x, PBP3, PBP4, PBP5 and PBP6 are analyzed and 19 β-lactam antibiotics are optimized to have minimal potential energy using chimera and then the virtual screening study is carried out for ligand molecules. From the virtual screening analysis, we list binding mode of Penicillin derivatives and Cephalosporins based on total energy (Table 2). The best binding poses for each ligand molecule into each target protein are determined and the one having lowest binding energy among the different poses generated. The lower energy scores represent better protein–ligand binding affinity compared to higher energy values. Among the 19 ligands, Cephalosporins are found to have lower binding energy value than the Penicillin derivatives. Especially the fifth generation Cephalosporins, Ceftaroline and Ceftobiprole has least binding energy value. Ceftobiprole shows best binding pose with PBP1b, PBP2a, PBP2b and PBP2x (total energy value for PBP1b = −110.7 kcal/mol, PBP2a = −108.2 kcal/mol, PBP2b = −110.4 kcal/mol, PBP2x = −116 kcal/mol). The Ceftaroline shows best binding conformation with PBP3, PBP4, PBP5 and PBP6 (total energy for PBP3 = −114 kcal/mol, PBP4 = −104.8 kcal/mol, PBP5 = −131.2 kcal/mol and PBP6 = −118.0 kcal/mol). On comparing the binding mode of Penicillin derivative, Azlocillin shows higher binding affinity with PBP1a (total energy value = −122.1 kcal/mol). These compounds have more stable ligand–receptor complex amongst other compounds. We further analyzed the docked conformation for finding the binding mode of fifth generations Cephalosporins, Ceftaroline and Ceftobiprole into selected target proteins to validate the position obtained likely to represent reasonable binding modes or conformations.

**Table 2:** Virtual screening results of β-lactam antibiotics by iGEMDOCK

S. no	#Ligand	PBP-1A	PBP-1B	PBP-2A	PBP-2B	PBP-2X	PBP-3	PBP-4	PBP-5	PBP-6
1	Amoxicillin	−121.1	−103.7	−89.8	−88.2	−84.1	−94.5	−67.6	−98.7	−79.9
2	Ampicillin	−89.3	−69.3	−87.3	−76.1	−82.1	−86.0	−64.8	−94.4	−84.4
3	Azlocillin	−**122.1**	−83.3	−99.8	−93.9	−84.4	−100.4	−72.9	−91.4	−85.0
4	Carbenicillin	−86.6	−74.9	−91.8	−84.7	−97.6	−98.2	−75.5	−107.6	−90.4
5	Cefadroxil	−107.5	−87.4	−90.1	−100.3	−101.7	−112.9	−72.3	−88.6	−83.4
6	Ceftobiprole	−104.6	−**110.7**	−**108.2**	−**110.4**	**−116.0**	−113.0	−83.2	−113.0	−102.0
7	Ceftaroline	−104.3	−97.1	−79.4	−97.0	−104.4	**−114.1**	**−104.8**	**−131.2**	**−118.0**
8	Cefuroxime	−103.4	−104.0	−94.6	−110.3	−94.3	−86.1	−78.2	−91.0	−80.0
9	Cloxacillin	−91.4	−90.9	−83.3	−89.4	−82.0	−89.4	−69.0	−94.3	−82.0
10	Dicloxacillin	−95.9	−83.9	−85.7	−82.6	−99.1	−97.1	−62.7	−85.2	−84.0
11	Flucloxacillin	−89.6	−75.4	−96.6	−88.9	−74.9	−89.8	−62.1	−90.8	−82.1
12	Lactivicin	−91.1	−95.9	−87.1	−90.9	−95.8	−95.3	−65.6	−98.1	−89.0
13	Methicillin	−95.5	−102.1	−102.0	−97.0	−92.3	−109.6	−74.9	−93.5	−94.0
14	Mezlocillin	−88.6	−101.9	−102.1	−92.9	−97.4	−105.0	−72.4	−112.1	−97.1
15	Nafcillin	−89.3	−100.8	−77.3	−82.0	−111.4	−101.5	−67.1	−97.8	−84.3
16	Oxacillin	−101.6	−88.5	−94.2	−86.9	−78.1	−90.3	−67.5	−97.4	−87.1
17	Penicillin G	−84.3	−74.4	−77.3	−79.3	−83.1	−89.5	−66.2	−81.6	−72.2
18	Piperacillin	−89.2	−86.4	−81.3	−97.5	−97.1	−103.7	−73.4	−99.8	−88.0
19	Ticarcillin	−99.8	−79.1	−81.6	−85	−80.9	−86.3	−64.7	−95.2	−92.0

The values in bold font indicate best binding energies

### Docking of Ceftobiprole into PBPs

**Fig. 2:**
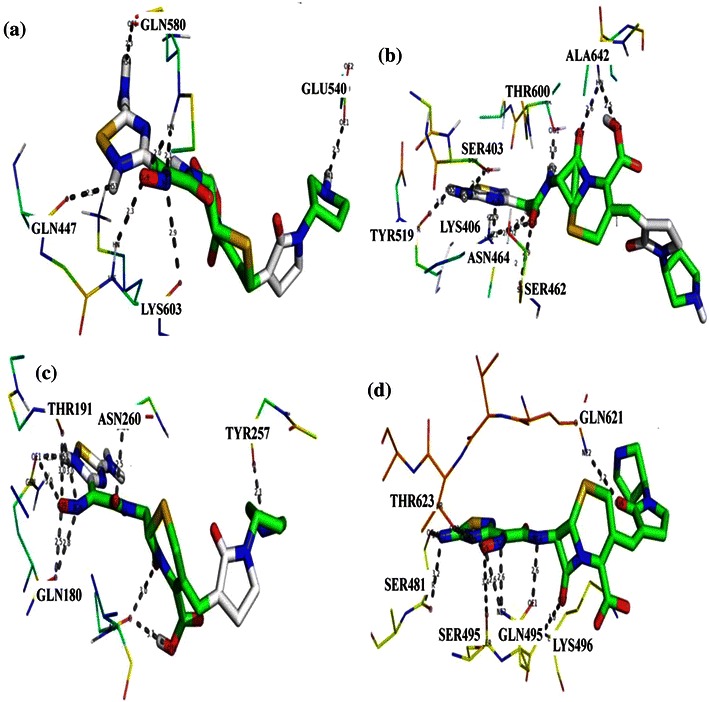
Docking results of Ceftobiprole against PBP1b, PBP2a, PBP2b and PBP2x. **a** Binding mode of Ceftobiprole in PBP1b. **b** A close-up view of the binding site of Ceftobiprole in PBP2a. **c** Ceftobiprole interaction with PBP2b. **d** Binding mode of Ceftobiprole with PBP2x. Ligand atoms are *colored* by its type. The interacted amino acids residues, hydrogen bond networks in the binding pocket and the distance (in Å units) of bonds are all shown

Docking simulation of Ceftobiprole is performed for PBP1a, PBP1b, PBP2a, PBP2b, PBP2x, PBP3, PBP4, PBP5 and PBP6. From the docking result, we identified that Ceftobiprole has best binding affinity with the PBP2x of *S. aureus.* Docking of Ceftobiprole results in the formation of more than five hydrogen bonds with PBP1b, PBP2a, PBP2b and PBP2x (Fig. 2). Amino acid residues Gln582, Glu540, Lys603 and Gln601 are involved in interaction with PBP1b; in PBP2a, the interacting amino acids are Ala642, Thr600, Tyr519, Ser403, Ser462, Asn464 and Lys406. In PBP2b, Asn260, Tyr257, Thr191 and Gln180 are involved in the interaction with Ceftobiprole. In close assessment of this binding mode, binding docking energies are calculated for PBP1b, PBP2a, PBP2b, and PBP2x (Table 3). In PBP2x, the amino acid residues Gln621, Lys496, Gln495, Ser481 and Thr623 interact with Ceftobiprole (Table 4). Davies et al. (2006) report that Ceftobiprole itself inhibits PBP1a, PBP2b and PBP2x, which are responsible for Penicillin resistance in *S. pneumoniae*. Our results are similar to the findings of Davies et al. Ceftobiprole, a fifth generation Cephalosporin in phase 3 clinical trials, exhibits a broad spectrum of activities against many clinically important Gram-positive and Gram-negative pathogens, such as *S. pneumoniae*, *H. influenzae*, and *S. aureus* (Hebeisen et al. 2001; Jones et al. 2002; Kosowska et al. 2005; Zbinden et al. 2002). Docking analysis of Ceftobiprole shows best results against *S. pneumoniae* and *S. aureus*. Our results are similar to previous studies (Hebeisen et al. 2001; Jones et al. 2002; Kosowska et al. 2005). Lovering et al. (2012) report that the affinity of Ceftobiprole to PBP2a of MRSA is high. Henry et al. (2010)

 report that PBP5 has less sensitivity to Ceftobiprole than PBP2a. Another study reveals that Ceftobiprole is a novel broad-spectrum antibiotic that inhibits PBP2a and PBP2x, which are responsible for the resistance in *S. pneumoniae* and *S. aureus*, respectively (Dauner et al. 2010). Though many reports on the inhibitory activity of Ceftobiprole for specific PBPs are available in literature, none of the studies have focused on the binding pattern of Ceftobiprole to all type of PBPs. Our study reveals the binding pattern of Ceftobiprole with all type of PBPs. The possible binding mode of Ceftobiprole in the PBP1b, PBP2a, PBP2b, PBP2x binding site and corresponding 2D interaction models along with hydrogen bonds and bond distance are shown in Fig. 2.

**Table 3:** AutoDock estimated docked energies of Ceftobiprole and Ceftaroline

S. no	Target	Ceftobiprole (kcal/mol)	Ceftaroline (kcal/mol)
1	PBP1a	−5.1	−5.2
2	PBP1b	−6.76	−4.12
3	PBP2a	−6.12	−3.43
4	PBP2b	−7.04	−5.1
5	PBP2x	−7.32	−5.3
6	PBP3	−6.1	−7.42
7	PBP4	−4.34	−5.65
8	PBP5	−6.21	−9.2
9	PBP6	−5.3	−8.3

**Table 4:** H-bond interactions and bond length obtained for Ceftobiprole with PBP1b, PBP2a, PBP2b and PBP2x

Protein–ligand complex	H-bond interactions	Bond length (Å)
Ceftobiprole-PBP1b	(Gln 582)O-H54	2.3
	(Gln 582)NH-029	2.0
	(Gln 582)NH-N28	2.1
	(Glu 540)O-H41	2.5
	(Lys 603)O-N28	2.9
	(Lys603)NH-O29	2.3
Ceftobiprole-PBP2a	(Ala642)NH-O34	2.5
	(Ala642)NH-O17	2.6
	(Thr600)O-H52	1.8
	(Tyr 519)O-H55	1.8
	(Ser403)O-H54	2.1
	(Ser462)O-O20	2.9
	(Asn 464)H-O29	2.0
	(Lys 406)H-O20	2.8
Ceftobiprole-PBP2b	(Asn260)H-O20	2.5
	(Tyr257)O-H41	2.1
	(Thr191)O-O29	3.0
	(Thr191)O-N28	3.0
	(Gln180)O-O29	3.0
	(Gln180)O-N28	2.8
Ceftabiprole-PBP2x	(Gln621)N-O36	3.2
	(Lys496)N-O17	3.0
	(Gln495)O-N18	2.6
	(Gln495)N-N28	2.8
	(Gln180)N-O29	2.6
	(Ser 495)O-O29	3.4
	(Thr 623)O-O29	3.2
	(Ser 481)O-N27	3.2

### Docking of Ceftaroline into PBPs

**Fig. 3:**
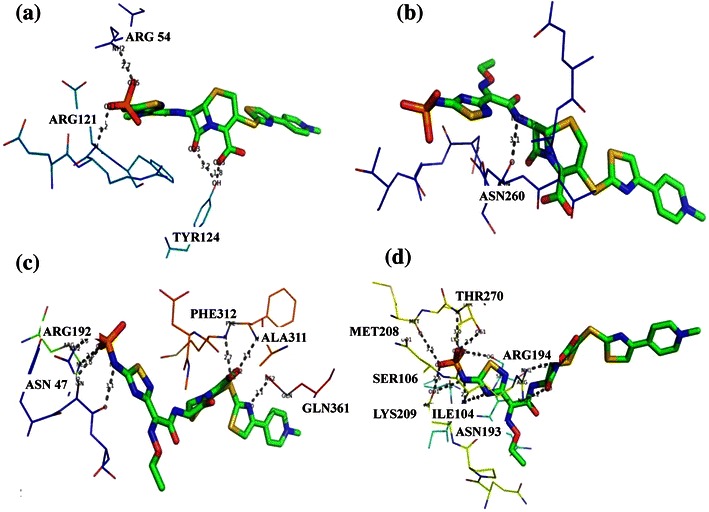
Docked complex of Ceftaroline–PBP3, PBP4, PBP5 and PBP6. **a** A close-up view of the predicted binding site for Ceftaroline in PBP3. **b** Binding mode of Ceftaroline with PBP4. **c** Ceftaroline binding site in PBP5. (3D) Interaction of Ceftaroline with PBP6. Ligand atoms are *colored* by its type. The interacted amino acids residues, hydrogen bond networks in the binding pocket and the distance (in Å units) of bonds are all shown

Ceftaroline is a antibiotic of the Cephalosporin type among the majority of currently available β-lactam antibiotics. Cephalosporins are used for effective treatment of bacterial respiratory tract infections. In our results on the binding conformation modes of Penicillin derivatives and Cephalosporins with PBPs, Ceftaroline shows higher affinity with the PBP3, PBP4, PBP5 and PBP6 than the other PBPs. In examining the interaction and position of the Ceftaroline in PBP3, PBP4, PBP5 and PBP6 active site predicted by our docking procedure, it is observed that multiple hydrogen bonds are formed (Table 5). In addition, the amino acid residues Arg54, Glu121 and Tyr124 of PBP3 are involved in van der Waals’ interactions. In PBP4, only one amino acid residue Asn260 is involved in interaction with Ceftaroline. Binding of Ceftaroline to PBP5 and PBP6 involves more than six hydrogen bonds. The binding affinity of Ceftaroline for MRSA PBP2a, methicillin-susceptible *S. aureus* (MSSA) PBPs 1 to 3, and *S. pneumoniae* PBP2x/2a/2b correlates well with its low MICs and bactericidal activity against these resistant organisms (Kosowska et al. 2010; Moisan et al. 2010). Citron et al. report the effects of Ceftaroline activity against Gram-positive and Gram-negative pathogens, including MSSA, MRSA, *E. faecalis*, *S. pyogenes*, *S. pneumoniae*, *H. influenzae*, *M. catarrhalis*, *K. pneumonia*, *E*. *coli*, *P. aeruginosa*, and *A. baumannii* (Citron and Goldstein 2008; Jones et al. 2005). Other studies reveal that Ceftaroline has potent activity against MRSA and *S. pneumoniae*. The Gram-negative spectrum of Ceftaroline is similar to that of other broad-spectrum Cephalosporins (Estrada et al. 2008; Moisan et al. 2010; Kosowska et al. 2010). Morrissey et al. report that the Ceftaroline has excellent activity against MRSA and Penicillin-resistant *S. pneumoniae*. Furthermore, Ceftaroline maintains good activity against *H. inlfuenzae* (Sader et al. 2005; Mushtaq et al. 2007; Morrissey et al. 2009). Our results are consistent with the previously studied ones (Kosowska et al. 2010; Moisan et al. 2010; Citron and Goldstein 2008; Jones et al. 2005; Estrada et al. 2008; Kosowska et al. 2010; Sader et al. 2005; Mushtaq et al. 2007; Morrissey et al. 2009). Although many studies have been reported the inhibitory action of Ceftaroline to specific PBPs, no studies have been done for the binding pattern of Ceftaroline with all type of PBPs. Our results clearly explain the binding pattern of Ceftaroline with all type of PBPs. The binding energy calculated by AutoDock for Ceftaroline–PBP complexes is shown in Table 3. The best possible binding mode of Ceftaroline in PBP4, PBP5 and PBP6 and their corresponding 2D interaction models are displayed in Fig. 3.

**Table 5:** H-bond interactions and bond length obtained for Ceftaroline with PBP3, PBP4, PBP5 and PBP6

Protein–ligand complex	H-bond interactions	Bond length (Å)
Ceftaroline-PBP3	(Arg54)NH-O15	2.7
	(Gln121)N-O13	3.2
	(Tyr124)OH-O43	1.8
	(Tyr124)O-O23	3.2
Ceftaroline-PBP4	(Asn260)O-N18	3.1
Ceftaroline-PBP5	(Ala311)N-O42	3.1
	(Gln366)N-N30	3.1
	(Phe312)N-O43	2.7
	(Arg192)N-O15	2.8
	(Asn47)N-O13	2.6
	(Asn47)O-N11	3.1
Ceftaroline-PBP6	(Thr270)N-O14	3.0
	(Thr270)O-O14	2.7
	(Arg194)NH-O43	3.2
	(Arg194)NH-O23	3.2
	(Asn193)N-N10	3.4
	(Asn193)O-N11	3.1
	(Asn193)O-O13	2.6
	(Ile104)O-N11	2.7
	(Met208)O-O13	3.5
	(Lys209)N-O15	3.0
	(Ser106)O-O5	3.1

### ADME screening

For each of the Penicillin derivatives and Cephalosporins, we analyzed for a number of physiochemical properties and pharmaceutically relevant properties, such as molecular weight, H-bond donors, H-bond acceptors, logP (octanol/water), and their position according to Lipinski’s rule of 5 (Table 6). Lipinski’s rule of 5 is a rule of thumb to predict drug likeness, or determine if a compound with a certain biological or pharmacological activity has properties that would make it a likely orally active drug in humans. The rule describes physiochemical properties important for a drug’s pharmacokinetics in the human body, including its ADME. The drug molecule shows poor absorption and permeation when they have more than 5 hydrogen bond donors, molecular weight over 500, logP is over 5 and more than 10 hydrogen bond acceptors. In this study, of the 19 ligands, 16 structures showed possible values for the properties analyzed and exhibited drug-like characteristics based on Lipinski’s rule of 5. Methicillin has more than 7 rotatable bonds. Rotatable bond more than 10 and molecular weight more than 500 can lead to decreased permeability and oral bioavailability. But Ceftobiprole and Ceftaroline show molecular weight more than 500. Hence to improve the action of these two drugs, we have highlighted the non-essential regions (Fig. 1) that may possibly be spliced to reduce the molecular mass. However, the effectiveness of these low molecular mass compounds has to be tested in both in vivo and in vitro.

**Table 6:** Molecular properties of Penicillin derivatives and Cephalosporins obtained from Molinspiration

S. no	Antibiotics	LogP (<5)	Molecular weight (<500 dalton)	HBA count (<10)	HBD count (<5)	Rotatable bond count (<7)
1	Amoxicillin	2.31	365.40	6	4	4
2	Ampicillin	−2.00	349.40	5	3	4
3	Azlocillin	0.20	461.49	6	4	5
4	Carbenicillin	1.13	378.39	6	3	5
5	Cloxacillin	2.61	435.88	5	2	4
6	Dicloxacillin	2.90	470.32	5	2	3
7	Flucloxacillin	2.69	453.87	5	2	3
8	Methicillin	0.85	380.41	10	3	11
9	Mezlocillin	0.21	539.58	8	3	5
10	Nafcillin	3.21	414.47	5	2	5
11	Oxacillin	2.05	401.43	5	2	4
12	Penicillin G	1.5	334.39	4	2	4
13	Piperacillin	1.2	517.55	7	2	6
14	Ticarcillin	0.99	384.42	6	3	5
15	Ceftobiprole	−1.68	564.16	11	7	4
16	Ceftaroline	2.43	699.03	16	5	2
17	Cefadroxil	−1.22	377.10	7	5	3
18	Lactivicin	−0.60	296.14	5	1	2
19	Cefuroxime	−0.2	424.39	10	3	7

## Conclusion

In the present study, molecular docking studies were performed to explore possible binding modes of Penicillin derivatives and Cephalosporins into all types of PBPs, PBP1a, PBP2b, PBP2x and PBP3 of *S. pneumoniae,* PBP1b, PBP2a and PBP4 of *S. aureus*, PBP5 of *H. influenzae,* as these organisms are most frequently found pathogens in the URT. The molecular docking study revealed that the Cephalosporins show higher affinity with PBPs than the Penicillin derivatives. Especially the fifth generation Cephalosporins, Ceftobiprole and Ceftaroline show best results to all types of PBPs. The binding affinity was evaluated by the binding free energies (DGb, Kcal/mol) and hydrogen bonding. The compounds which revealed the highest binding affinity are the ones with lowest binding free energy. On comparing the binding energy and the binding site residues, we found that all compounds differ in their binding modes or binding site residues for hydrogen bond formation. The conclusion drawn from this virtual screening and docking result was that the Ceftobiprole has highest binding affinity with the PBP2x of *S. pneumoniae.* The Ceftaroline has maximum number of interaction with PBP5 of *H. influenzae.* The above results suggest that the Ceftobiprole and Ceftaroline can be potent inhibitors for all types of PBPs. From ADME screening of all the 19 compounds, 16 compounds satisfied Lipinski’s rule of 5. Ceftobiprole and Ceftaroline show molecular weight more than 500 which decreases their permeability and bioavailability. These drugs can further be modified to satisfy Lipinski’s rule of 5. Though, there are a few reports on the in vitro analysis of Ceftobiprole and Ceftaroline, there are no in silico studies that predict the binding and active regions in these molecules. Our study is probably the first such attempt and we infer that our results will throw light for the future development of more potent next generation antibiotics for the treatment of upper respiratory infections and counter the emergence of antibiotic resistant strains.
